# Surviving the Wildlife Trade in Southeast Asia: Reforming the ‘Disposal’ of Confiscated Live Animals under CITES

**DOI:** 10.3390/ani11020439

**Published:** 2021-02-08

**Authors:** Shannon N. Rivera, Andrew Knight, Steven P. McCulloch

**Affiliations:** Centre for Animal Welfare, University of Winchester, Winchester SO22 4NR, UK; Andrew.Knight@winchester.ac.uk (A.K.); Steven.McCulloch@Winchester.ac.uk (S.P.M.)

**Keywords:** CITES, wildlife disposal, animal welfare, conservation, wildlife confiscations, illegal wildlife trade

## Abstract

**Simple Summary:**

In response to the illegal wildlife trade, successful enforcement often involves the seizure, confiscation, and subsequent management of illegally traded wildlife. Unfortunately, confiscated live animals often experience substandard care. In this study, we investigate the barriers to the ‘disposal’ of confiscated live animals in Southeast Asia. ‘Disposal’ is the term used for what happens to illegally traded wildlife after confiscation. Guidelines for the ‘disposal’ of live specimens are provided by the Convention on the International Trade in Endangered Species of Wild Fauna and Flora (CITES), although individual nations must enforce this within their own legislation. We interviewed 18 experts from seven countries in Southeast Asia revealing eight categories of barriers to the disposal of confiscated live animals. We then propose seven recommendations to help reform the disposal of confiscated live animals, which would support the efficient and humane management of illegally traded wildlife in Southeast Asia and globally.

**Abstract:**

Increased focus on the illegal global wildlife trade has resulted in greater numbers of live animals confiscated by authorities, increasing the need to manage these animals responsibly. Most wildlife seizures take place in Southeast Asia, with global demand for live animals fuelling much of the trafficking. Guidelines for the ‘disposal’ of live specimens are provided by the Convention on the International Trade in Endangered Species of Wild Fauna and Flora (CITES), although individual Parties must implement provisions through national laws and regulations. ‘Disposal’ is the term used for the management of illegally traded wildlife upon confiscation. Confiscated live animals can be euthanised (i.e., killed), repatriated to their native country and released, or kept in captivity. This study investigates barriers to proper care and disposal of confiscated live animals in Southeast Asia, where roughly one quarter of the global multibillion dollar illegal wildlife trade takes place. Interviews were conducted with 18 professionals working within conservation, wildlife crime, and confiscated live animal management. Eight limitations to the proper care and disposal of confiscated wildlife were identified: (1) political will, (2) policy, (3) funding, (4) capacity, (5) expertise (6) attitudes and behaviours, (7) exploitation, and (8) corruption. Based on interviews, we propose seven key reforms to support the efficient and humane management of illegally traded wildlife for national authorities and CITES parties. These are wildlife seizure management, legislative support, enhanced political will, demand reduction, global participation, registry of rescue centres, and terminology change. This research highlights major barriers to the proper care and disposal of live confiscated animals and proposes key reforms to improve the conservation of threatened species and the welfare of millions of illegally traded animals.

## 1. Introduction

The illegal wildlife trade has seen unprecedented rates of growth escalating the trade into an international crisis [1,2]. In response, there has been a concerted international effort to stop the illegal wildlife trade, resulting in increased laws, regulations, enforcement, and interception [3,4]. Wildlife seizures have become a globally recognised approach in disrupting illegal markets [2,4]. These changes have resulted in an increase in the number of live wild animals that are confiscated by government agencies [4,5] thereby elevating the need to manage and ‘dispose’ of these animals humanely and responsibly. ‘Disposal’ is the technical term used to describe the management of illegally traded and confiscated live animals. Alternative terms are being considered [6], however this study uses the term ’disposal’ to be consistent with existing internationally recognised terminology and guidelines.

Live animals are an important component of the industry in both legal and illegal markets. Live animals are traded to meet consumer demand for pets, wild meat, working animals, entertainment, and as status symbols [7]. Hundreds of different species from dozens of taxonomic groups are trafficked live [8], however available seizure data shows that the most commonly trafficked live animals are overwhelmingly reptiles for use in the pet trade [8,9]. Based on seizure data, Asia is the main destination for the illegal live reptile trade [2]. The trafficking of live animals threatens the welfare of individual animals, local populations, and the extinction of whole species [10] while degrading ecosystems [11]. The live animal trade is also a major channel of disease introduction worldwide threatening livestock, native wildlife, ecosystems, and also people [4,5,6,7,8,9,10,11,12]. The ‘exotic’ pet trade is a substantial part of the global trade in wildlife and has been identified as an important driving factor in the emergence of zoonotic diseases [13]. The global coronavirus (COVID-19) pandemic of 2020, for example, has been epidemiologically linked to a wet market in Wuhan, Hubei Province, China, which sold both wild and domesticated live animals in close proximity [14]. Furthermore, the live animal trade also has particular significance due to the prolonged negative impacts on animal welfare [10,13,15].

The Convention on International Trade in Endangered Species of Wild Flora and Fauna (CITES) is a multilateral treaty established to ensure international trade in wildlife does not threaten the survival of species [16]. The Parties adopted Resolution 17.8, ‘Guidelines for the Disposal of Confiscated Live Specimens of Species included in the Appendices’ [6], to provide guidance on the issue of disposal. This resolution includes three accepted disposal options for living specimens: return to the wild, captivity, and euthanasia. 

Legislation, cultural practices, and economic circumstances influence decisions on appropriate disposal of confiscated animals [4,17]. Parties are instructed to obtain advice from their own Scientific Authority when making the decision on the disposal of confiscated live specimens [18]. According to CITES, the ultimate decision must achieve three goals:

1. to maximize conservation value of the specimens without in any way endangering the health, behavioral repertoire, or conservation status of wild or captive populations of the species;
2. to discourage further illegal or irregular trade in the species; and
3. to provide a humane solution, whether this involves maintaining the animals in captivity, returning them to the wild, or employing euthanasia to destroy them [18].

It is important to note that there is a significant number of non-CITES listed species that are traded internationally and domestically [19]. For instance, fewer than 8% of the total number of reptile species are regulated by CITES [20]. However, a 2020 study found that 35% of the 10,272 currently recognised reptile species are traded in the pet market with 90% of species being captured from the wild, predominantly from hotspots within Asia [21]. Seventy-five percent of reptile species traded were not covered under international trade regulations [21], which means it is more difficult to intercept these species through confiscation. This creates opportunities for exploitation of species that are threatened but still legally traded domestically, or internationally without data to support the trade impacts to the species. 

Authorities have a responsibility to dispose of confiscated live animals appropriately, however, data on outcomes is lacking [8]. This impedes efforts to effectively monitor and evaluate management outcomes. To address this, CITES investigated how parties are managing disposal of live specimens in 2016 [6]. The study found that over 86% of reported confiscated live animals were placed in captive facilities [22], but many of these facilities are not formally regulated at a standardised capacity creating avenues for exploitation and poor conditions [23,24]. 

In the absence of appropriate legislation, well-defined enforcement protocols, and comprehensive records of illegal trade activity, it is not possible to judge a state’s commitment to controlling the illegal wildlife trade or preventing animal welfare violations. Despite the potential for recovering illegally traded live animals through confiscation and replenishing endangered wild populations [25], providing proper management for confiscated live animals is an overlooked aspect in the global response to this trade [26]. Focusing attention on a region that serves as a source, transit, and destination for the wildlife trade may facilitate understanding of live animal confiscation cases around the world. This study aims to improve understanding of the management of confiscated live animals by qualitatively investigating the limitations impeding proper disposal methods in Southeast Asia.

### 1.1. Illegal Wildlife Trade

Wildlife crime is defined as ‘the taking, trading, exploiting or possessing of the world’s wild flora and fauna in contravention of national and international laws’ [27]. Wildlife trafficking is one of the most profitable illicit industries but despite global attention and increased policy against illegal sales, scientists have yet to describe the scope and scale of the trade [28]. The elusiveness of the trade makes it nearly impossible to accurately estimate its monetary value, but the International Criminal Police Organization (INTERPOL) estimates it is worth at least USD 20 billion a year [27].

It is known that wildlife trafficking is a leading threat to biodiversity impacting one-third of the world’s species [9,29]. Some studies have suggested that the existence of legal international trade enables the illegal trade in wild-caught animals [30,31]. For example, a legal quota system may facilitate illegal trade through deliberate or accidental misidentification of species and false permits [32,33]. Despite the international crisis, designation, regulation, and enforcement efforts still remain insufficient to effectively control this illicit trade [34] (p. 1), and with a globalised market driving demand, it continues to rapidly increase [17] (p. 2).

### 1.2. The Wildlife Trade in Southeast Asia

**Figure 1:**
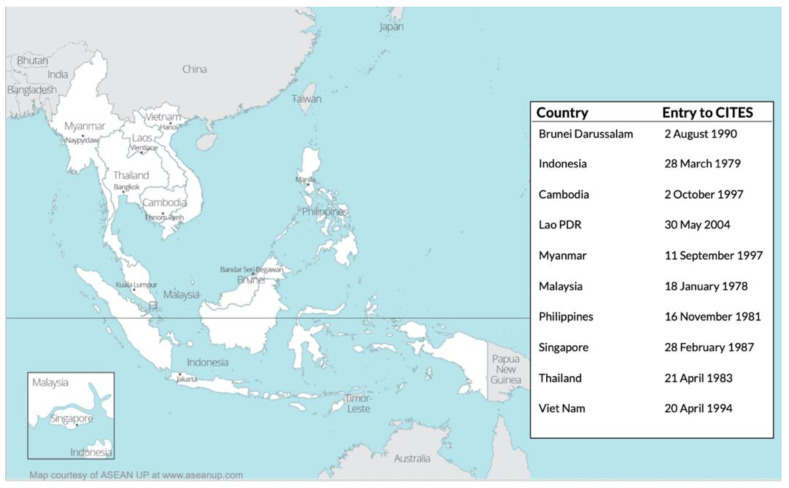
Southeast Asian countries and accession to the Convention on the International Trade in Endangered Species of Wild Fauna and Flora (CITES.) (Map courtesy of ASEAN UP via aseanup.com. CITES data retrieved from CITES.org).

Southeast Asia is a critical region of importance for the wildlife trade [35] (Figure 1). The region is responsible for roughly a quarter of the global multibillion-dollar illegal wildlife trade [32] with the demand for rare and exotic pets fuelling much of the smuggling [36]. The United States alone imported over 1.48 billion live animals in less than a decade, mostly from wild populations in Southeast Asia [37]. To illustrate, more than 100,000 pig-nosed turtles (*Carettochelys insculpta*) were seized in Indonesia alone from 2003–2019; and in just 10 incidents, more than 6000 Indian star tortoises (*Geochelone elegans*) were seized before transport to either Malaysia, Thailand, or Singapore [35]. A significant amount of global wildlife seizures also occurs in Southeast Asia [9,35]. This makes Southeast Asia particularly important when investigating live animal confiscations. Regional enforcement initiatives have been established in the region to respond to the growing issue [38]. As a result, wildlife traffickers have been intercepted at greater rates [39] (p. 5), creating unexpected management challenges of illegally traded and confiscated live animals [40]. Serious pressures have been placed on confiscating authorities, with many lacking the financial and human resources and the technical capacity to manage the situation adequately [5,41] (p. 4).

Legislation and increased regulation add to the burden of management challenges and can be a disincentive to conduct a seizure if resources and training are lacking [40,42]. Authorities have been reported to frequently release rescued animals to the wild without complying with proper procedures [42]. Documentation of the confiscation and essential details regarding the trade can be lost [43]. Animal laundering schemes and the legal sale of valuable confiscated specimens are also a concern [42]. There have been reports of confiscated animals sold to other countries and registered on annual reports as legal trades [44]. Selling confiscated animals can even comply with CITES guidelines through the justification that it can ‘provide a means of disposal that helps offset the costs of confiscation’ [18]. D’Cruze and MacDonald [8] have brought attention to the lack of comprehensive live animal confiscation records within the CITES Trade Database. They highlighted a knowledge gap and helped bring into question whether countries are properly handling live confiscations. Though the international efforts to stop the illegal wildlife trade have increased, with more live animals being successfully seized, in many cases, recovered individuals may face equally bad or even worse outcomes [24].

### 1.3. The ‘Disposal’ of Confiscated Wild Animals

Within a conservation context, there are three accepted options for proper disposal, with both conservation and welfare impacts to consider: (1) euthanasia, (2) return to the wild, and (3) captivity. The International Union for the Conservation of Nature (IUCN) considers euthanasia to be the preferred disposal option [40]. Arguably, however, the term ‘euthanasia’ does not apply to a large proportion of confiscated live animals. Euthanasia should be restricted to killing an animal for its own benefit rather than human convenience or need [45,46]. Such instances might include animals infected with a harmful disease or when adverse conditions will seriously affect an animal’s quality of life [4]. It is difficult to determine when true euthanasia has been implemented, therefore, this paper uses the broader term of ‘killing’ instead of ‘euthanasia’. The IUCN report that killing is the least risky and least costly of the three disposal options [40]. However, killing wild confiscated animals is often controversial. Furthermore, killing is not considered an option in many Southeast Asian countries because of religious and cultural reasons [4].

Repatriating and releasing confiscated and rehabilitated wildlife back into their natural habitat, by way of reintroduction, translocation, or reinforcement, would in many cases generate the best conservation and animal welfare outcomes; however, it comes with a high probability of risk [4,18]. Returning animals to the wild is actually the least recommended option in the handling of confiscated wildlife, and the IUCN have reported that it is not often done [18,40]. Still, returning confiscated and rehabilitated wildlife back into their natural habitat has the potential to be a powerful conservation tool if implemented correctly [47] and could ultimately produce population-level benefits [48]. Retaining confiscated wild animals in captivity for the remainder of their natural lives is the third option [4]. Like returning to the wild, captivity comes with risks such as disease or escape and financial costs [18]. Minimising the risks associated with releasing to the wild and captivity would require improvements and revisions to confiscation management practices [40,49], an investment that may be warranted.

At the 17th meeting of the CITES Conference of Parties, Decisions 17.118 and 17.119 directed an investigation of how Parties are disposing of illegally traded and confiscated specimens of CITES-listed species [6]. Results of the investigation were published as SC69 Document 34.1 [22]. Of the 183 countries party to CITES, 125 (68%) countries did not respond. From the 58 countries that did respond, over half (54%) indicated that they did not have an established plan of action for making decisions on a disposal method for confiscated live specimens [22,50]. The survey found that the majority of confiscated live animals are reportedly placed in captivity (43.6% public zoos, safari parks, aquariums, or botanical gardens; 27.4% designated rescue centres; 6% research institutes, laboratories, and universities; and 9.4% were placed in approved private facilities) [22]. This correlates with research identifying overcrowding as the most significant problem in Southeast Asian zoos due to the large number of confiscated and abandoned animals [51]. Only 6.0% were reported as killed, and it was reported that 6% of animals confiscated were returned to the country of export for release to the wild [22].

Captivity is the most commonly reported method of disposal, yet there are no standardised, enforceable requirements for captive facilities at the international level and few nations have laws that regulate the treatment of captive wild animals [52,53]. There are significant animal welfare impacts and violations associated with captivity that can negatively affect captive wild animals [54,55]. Captivity generally means wild animals are confined in spaces that are far smaller than their normal ranges. Animals are often kept in conditions that are very different than they have evolved to live in. Such different conditions can lead to stress and poor physical and mental welfare through a range of mechanisms. Public facilities that have a visitor element can negatively impact animals [56,57]. The mere presence of people has been shown to elevate levels of stress in captive animals [56,58,59]. This is particularly concerning with increasing numbers of wildlife tourists, such as those visiting rescue centres or viewing animals in the wild [60].

In areas where facilities are available, high intake numbers and a lack of funding make implementing a strategic conservation plan or gathering objective scientific data difficult [40,61]. Frequently, minimal educational requirements for staff result in a lack of on-site ecological and ethological knowledge and record-keeping is often inadequate [62,63,64,65]. With the CITES survey indicating that many countries transfer the majority of confiscated animals into captive environments, examining this disposal option is important with regard to understanding the impacts of captivity on wild animals, and also how it is affecting conservation objectives for threatened species. CITES Resolution 17.8, Article VIII.4.b states that live specimens shall be placed in a ‘rescue centre or “such other place as the Management Authority deems appropriate and consistent with the purposes of the present Convention’ [18]. The broad guidelines on facilities that qualify as acceptable for housing confiscated wildlife, oftentimes valuable animals, may put those animals at risk of exploitation. The lack of formal mechanisms coupled with other challenges increase the chance for confiscated animals to be exploited even after recovery [24]. Lopes et al. [44] highlighted that CITES is working toward improving records by revising their annual reports to include disposal data, but significant gaps remain.

## 2. Methods 

This study investigates the following research questions:

(1) What are the limitations impeding disposal of confiscated live animals and how are they related?
(2) What are the consequences that arise from insufficient disposal methods?
(3) What are the perceptions surrounding the disposal of confiscated live animals?

A qualitative methodology was used to explore the multidimensional nature of the research area. The research aim was to gain insights into the subject under investigation from individuals with good awareness and knowledge of the subject area, hence purposive sampling of participants was used [66]. Snowball sampling was also used whereby interviewees recommended potential candidates with the appropriate qualifications and experience.

### 2.1. Data Collection

A total of 18 participants were recruited based on expertise as professionals with knowledge of confiscated live animal management. In an effort to ensure confidentiality, participants were coded as C1–C18. Participants worked within seven countries in Southeast Asia: Thailand, Brunei, Malaysia, Indonesia, Vietnam, the Philippines, and Cambodia. The contributing participants represented a variety of expertise, position, geographic location, and professional agency. Each interviewee had 6–30 years of experience working in wildlife crime, conservation, and/or confiscated live animal management. They were drawn from six international non-governmental (NGO) or intergovernmental organizations, four local/national NGOs, two NGO rescue centres, two governmental rescue centres, one private rescue centre, two government agencies, and one university. Ten other individuals invited to take part in the study were unable to participate. Three of these individuals included employees who were otherwise represented through counterparts within the agency or organization. Four individuals were unreachable, and three individuals declined the request for participation; all seven representing commercial establishments. Hence, 64% (18/28) of invited individuals chose to participate.

Semi-structured interviews were used to gather data about the research area. Telephone interviews were used for 17 participants, owing to the international locations they were based in. The remaining interview (C3) was conducted in-person. Interviews were conducted from July to September 2018. Interviews lasted from 30 min to 79 min, with an average length of 39 min. A participant information sheet and consent form were provided to each participant via email. Interviews were audio-recorded using a hand-held Sony IC recorder and transcribed for analysis. To ensure accurate communication and to establish trustworthiness, participants were given the option to conduct a member check after the interview was conducted. Member checks afford participants the opportunity to review data for clarification. The interview guide included seven questions (Table 1), which were followed up with further questions depending on the expertise of the participant and the subject under discussion.

### 2.2. Data Analysis

Transcripts were coded by grouping sections into themes, or categories of similar meanings [67]. A thematic coding framework was designed based on the systematic classification process of identifying themes or patterns [68,69]. The initial codes were deductively defined based on existing literature and empirical data. Relevant statements from interviews were also coded with keywords deduced from theoretical assumptions built on early interviews. Lastly, new topics were identified using inductive coding. The coded responses were manually grouped and entered into a Microsoft Excel file with designated tabs for each theme. Each tab was systematically analysed, generating a rich description of perceptions according to identified limitations to, and consequences of, the disposal of illegally traded and confiscated live animals. The defined set of codes and emergent themes were developed by the first author and agreed with the second and third authors. To ensure confidentiality, identifying markers including personally identifying phrases and professional agency were withheld when reporting results. At the request of several participants, country identifications were not assigned to quotations and specific locations were withheld.

## 3. Results

Eight prominent limitations impeding proper disposal of illegally traded and confiscated live animals were identified. These are (1) political will, (2) policy, (3) funding, (4) capacity, (5) expertise (6) attitudes and behaviours, (7) exploitation, and (8) corruption. The interview discourse that led to the categorization of these broad limitations is summarised in Table 2. Exploitation and corruption were both recognised as limitations impeding proper disposal methods as well as consequences that come from improper management. Findings are illustrated with selected quotations from participants. The findings are reported in a logical order in relation to policy. For instance, political will and policy are considered before funding and capacity because political will and policy influence funding and capacity.

### 3.1. Political Will

It was recognised by 12 participants that several identified limitations, including lack of funding, lack of expertise, lack of capacity, corruption, and exploitation, could be ameliorated through increased political will. Environmental apathy within the region’s political sector was a common theme recognised by participants. For example, C18 noted, ‘I’ve sat-in-on who knows how many government budget planning meetings and they don’t even consider [the disposal of confiscated animals] an issue. Even if it’s brought up, they don’t put any money into this and very few countries do.’

The lack of global political will (outside of Southeast Asia) has been perceived as a limiting factor because many animals confiscated in Southeast Asia are not native to the region. When discussing a case involving confiscated wallabies (*Macropus* spp.), C9 explained, ‘we wrote a letter to Australia, but they gave no intention of wanting the animals back and we cannot release them here, so they stay in captivity.’

### 3.2. Policy

Both the lack of policy and the presence of policy were be viewed as a limitation by 12 participants. It was identified that some states are still working to draft legislation on the disposal of live specimens showcasing a lack of policy. Alternatively, introducing excessively demanding regulations for management of confiscated wildlife was recognised as a disincentive. As C3 noted, ‘there is also a disincentive for these regulations to be put in place. If [regulations were] enforced, the country is going to say, “we can’t do that” and they’re not going to want to confiscate anything. If they have these regulations in place for a top-of-the-line facility, the bar is set so high that nobody can approach it and there isn’t even a point.’

Retaining live animals as evidence in criminal cases has been identified as a policy that impedes proper disposal of confiscated animals. C9 explains the barrier using the following example: ‘All confiscated animals, especially if there is a pending case, we cannot release them back. We are still waiting for court clearance and it’s very slow in [our country]. The fastest court clearance I’ve received was about two years. So, most of the time, the received animals were already dead before even getting clearance. Like last year I received a subpoena asking me for the status of birds that were confiscated way back in 2008.’

### 3.3. Funding

All 18 participants confirmed lack of funding and present economic constraints to be a considerable limitation that impedes the proper disposal of confiscated live animals. Substantial operating costs for facilities associated with managing confiscated animals in captivity include operational costs, medical care, the cost of land, and animal husbandry. Participants recognised that several other limitations, including expertise, capacity, and exploitation are mitigated through increased funding. Interviews brought attention to the direct relationship between the lack of political will and the lack of funding for management of confiscated live animals. Several participants identified a relationship between lack of funding and exploitation of confiscated live animals. C17 identified transparency issues associated with funding within their country by saying, ‘the government has a budget for [management of confiscated animals], but in reality, we never get paid from the government.’

### 3.4. Capacity

Capacity limitations encompass the lack of personnel, technical mechanisms, limited space, equipment, and number of animals as identified by 17 participants. The lack of space was identified as being a capacity issue, but constraint was largely based on financial restrictions rather than the lack of physical space; it was also, therefore, recognised as a funding limitation. Inadequate record-keeping was identified as a significant capacity issue limiting proper management. Five participants working at captive facilities raised concerns about the sheer number of animals being confiscated, coupled with the lack of personnel and space. C4 presented this concern by saying, ‘We get on average three animals every day and most of them are injured’ (C9), whereas another participant shared, ‘some days you will go in and [the rescue centre] will have 70 new animals.’

Five participants established that lack of capacity creates avenues for corruption. Furthermore, capacity constraints were identified as a deterrent for confiscation authorities to conduct further wildlife seizures. C3 noted, ‘pretty soon the officers just want to go and work drug cases because there is a mechanism on how to handle those cases. How to administer this type of work is critical … [but] logistically their hands are tied. What do they do? A lot of time they take money. They would like to confiscate, but there is no mechanism in place … a lot of times what they take is more than the fine would have been anyway.’

Nine participants described multiple cases of confiscated animals being released to the wild, many without proper mechanisms and protocols. Such as with the following example: ‘There are shipments of tons, literally tons of snakes per shipment that are just being released. Tons of live monitor lizards that are just being dumped out and now birds, they’re releasing cockatoos and all kinds of stuff into [non-native region]. It’s just ridiculous. The wrong species are getting dumped all over the place’ (C18). Not all releases were reported as being erroneous; some facilities claim to be releasing rehabilitated confiscated animals back to their native habitat but are unable to publish data on their methods and practices due to lack of capacity and funding. 

### 3.5. Lack of Expertise

Sixteen participants confirmed lack of expertise as impeding the proper disposal for confiscated live animals. Two participants clarified that expertise was not a limitation in the programs they were involved in but recognised that the lack of expertise is an overall limitation in their region. The expertise limitation was found to be alleviated for programs that accept experienced volunteers. Lack of taxonomic knowledge was perceived as an expertise limitation. C18 gave the example, ‘taxonomy and the genetics work in [the region] still have a long way to go. I mean the Philippine pangolin was only described a few years back, so up until a few years ago, any pangolin seized in mainland Southeast Asia from the Philippines was released as Sunda pangolin, so the wrong species were being released in the wrong place.’

With regard to captive facilities, it was noted that levels of education of staff differ country by country. C6 shared, ‘there are few, sort of, private rescue centres … mostly run by enthusiastic amateurs who haven’t gotten much, you know, professional ability to care for wildlife.’

Data showed expertise to be a more pressing limitation for programs impacted by economic constraints. As an example, an internationally funded program housing confiscated animals identified their facility as having 25 keepers, rotating volunteers, several researchers, and a resident veterinarian. A self-funded private facility in a neighbouring country with roughly the same number of animals described their management structure as a family that cares for the animals; one man with his children to help clean the facility and a veterinarian that visits every weekend to check on the animals. Expertise was perceived as a limiting factor amongst all confiscating authorities, government-run rescue centres, and privately-owned establishments. As highlighted by C6 who said, ‘generally, the government will have one official employee, all of the other staff will be local hires, you know, just people who live in the local village and stuff like that. I don’t know any … centre with [veterinarians] on the staff.’

### 3.6. Attitudes and Behaviours

The presence of detrimental attitudes and behaviours was identified as a limitation by 13 participants. Attitude and behaviour limitations include ego, cultural views, public perceptions, and institutional perceptions. Ego was identified by seven participants as being a constraint in the management of confiscated live animals. Ego-dominated managerial traits and structures impact proper care of confiscated animals. An incident was described where ego-driven opposition led to hundreds of confiscated endangered animals dying. C4 recalled the event by sharing, ‘one thing we tried to install was to just send animals, since specialist centres exist, to get them transferred to a specialist centre. We had a lot of opposition from the director because that’s very much the admission of being unable to look after them. Most of the animals died.’

Seven participants highlighted that ego-dominated decision-making regarding reintroduction failures and other challenges are underreported because of the potential repercussions. C6 captured this by stating,

there are massive reintroductions going on … [Those reintroducing] are a bit guarded on what they do because sometimes it goes wrong and they don’t want people to know. But negative data is definitely valuable data, you know, it can stop people making the same mistakes. It is important to get that out there, but I think many people are worried about repercussions from that. Especially inside the [government] … If a director makes a decision to do it and then there was a problem- that would affect [their] promotions and things like that.

Following rather than meeting needs was identified as often driving priorities for groups working with confiscated wildlife, at the cost to the animals. Trends included unnecessary and/or ineffective capacity building, the focus on charismatic species and, prioritizing volunteer programs focused on popular species. C10 shared, ‘rescue centres are full of species [that are commonly seized] and they don’t want anymore. You know, there’s too many of them flooding the rescue centres. The ones that aren’t flashy can’t raise money to pay for themselves to stay in the rescue centres, so what happens to them?’

Cultural values, religious beliefs, and public perceptions were recognised as a factor impeding confiscation management. Cultural views were perceived as a hindrance to the consideration of killing confiscated animals. Five participants noted that decision makers will rarely allow euthanasia even if it means that animals will suffer. Two admitted that they may allow it under extreme circumstances but will not take the action themselves. Perceptions on the status of animals amongst keepers, authorities, and the public are also included in this limitation. C4 shared, ‘there’s a fundamental gap… there’s only so much training you can do, like with so many of the [keepers]: an animal is seen as an object, a possession rather than a living thing.’ C9 noted, ‘most of [the police] don’t even care…when they see a deer, they see food … it’s the culture.’

Unsound releases were related to improper protocols as well as apathetic attitudes. Releasing confiscated animals into the wild was described as ‘throwing away’ unwanted responsibilities. C18 described an incident, ‘there was a recent seizure of 6000 birds in [Southeast Asian country]; 2000 died immediately and the other 4000—they were all just released in one spot. They don’t even know what species they were or where they were from. There was no quarantine process. Well, they said they were in quarantine, but for like a day, so it’s really a joke.’

Two participants claimed that negative attitudes towards specific species, such as snakes, restrict those species from being released back to the wild forcing them to remain in captivity or be euthanised when they are fit to be released. Perceptions within the conservation community were also identified as a limiting factor impeding proper disposal management.

### 3.7. Exploitation 

Exploitation has been recognised as both a limitation to proper disposal and a consequence of improper disposal by 16 participants. For the purpose of this study, exploitation is considered the act of making use of and/or benefiting from confiscated animals. Acknowledgment of exploitation predominantly focused on the use of confiscated animals in ‘for-profit’ schemes. The lack of clarification within international guidelines opened doors for exploitation. C12 shared that their country ‘sends confiscated animals to zoos, sanctuaries, and also tourist centres with personal owners … CITES allows for confiscated animals to be commercial, exhibition, to be sold, etc. and [some people] abuse that.’ Concerns were raised about facilities that housed ‘less popular’ common species receiving less funding and being in worse condition while facilities housing “popular” animals would keep them in captivity to maintain funding even when they had the potential to be released.

It has been identified that the demand for animal tourism has created a new market for confiscated wildlife. This demand is deterring possible rehabilitation and releases. C6 highlighted this by sharing, ‘when the animals come into these centres, when the centres start generating money, they fall into a sort of a corner where they actually don’t want to rehabilitate the animals … they make no attempt to release them because know if they managed to rehabilitate and release them, they’d have nothing for the tourists to see.’ C17 noted that ‘the first choice is supposed to be the rescue centre, but in reality, usually the ones that come to the rescue centre are the species that the zoo that didn’t want … the zoo almost never does any release because [the animals] are assets for them.’

The demand for animal tourism is creating faux-rescue centres and increasing the number of unregulated captive facilities. C6 provided the example, ‘the latest thing in [my country] is elephant rescue centres. There are no laws relating to them, so they are popping up all over the place … Instead of rescuing elephants, they’re buying elephants … the [sellers] use the money to buy a new elephant, they buy a young one and put it into the trade.’ C3 shared, ‘there is a lot of back door sales. Individual employees can make [USD] 10 grand cash for a baby tiger, that’s a 20-years salary. I can tell you that will happen in the United States too.’

Exploitation was not only recorded at public facilities; it was identified to occur within government agencies and reputable NGOs as well, as highlighted by an example from C14, ‘in the [fisheries agency], I’ve seen [exploitation] in their office, they [had] piranhas that were confiscated, but then they just keep it in the office. So, I don’t know their protocol on proper disposal.’ Additionally, C6 said,

some NGOs have a lot of integrity and are doing the right thing, but a lot of them are doing exactly the opposite and just totally exploiting the animals in another way. [Exploitation] is a very serious problem and it’s something that, many [NGOs] are aware of this problem but they don’t want to draw attention to it because it might negatively affect their business.

### 3.8. Corruption

Corruption has been recognised as both a limitation to proper disposal and a consequence of improper disposal by 10 participants. Corruption can be considered as dishonest or fraudulent conduct by those involved in the management of confiscated animals. Corruption was identified to infiltrate all levels of confiscation management from the government and political sphere to captive facilities and international players. Corruption has been identified as sometimes being a result of the lack of funding, capacity, and expertise. C3 noted ‘it’s not so much that they are corrupt but if they are supposed to seize animals that need to be immediately returned or cared for and there is no help … they don’t have a mechanism … so it ends up being corrupt. [Some] corruption is a result of trying to cut the best outcome out of a set of unpleasant circumstances.’

Findings uncovered that corruption and exploitation overlap, especially in the case of selling confiscated animals which is legal in many countries. C4 shared that ‘the enforcement officers … [may] auction the animals off, so they go back to the same sellers. People are telling me that it is happening. I’ve not witnessed it first-hand.’ C9 provided another example of corruption by sharing that in their country ‘the law in wildlife is so strong … the problem is the will and the enforcement, even the police, they would just whisper “I want my cut” … Corruption happens and then they always use ignorance, that they don’t know any better; it’s normal.’

The need to hold live animals as evidence was found to also create opportunities for corruption. C18 highlighted this by describing this experience:

I was part of a big seizure, the largest seizure ever for [a critically endangered animal] and the government’s stance was that they had all died, but everybody knows that they just got sold out the back door and that happens a lot … The government said they couldn’t send the live animals back to [country of origin] because they needed them as evidence for the case. So, when they claimed that they had died, I said, ‘well, surely you kept [them], you still need evidence for the case.’ And they said, ‘Well, no, no, of course not. We threw them all away.’ Which throws the whole story of needing evidence at the window.

In Table 3 below, the responses of individual participants relating to limitations impeding the proper disposal of confiscated live animals are summarised.

## 4. Discussion

**Figure 2:**
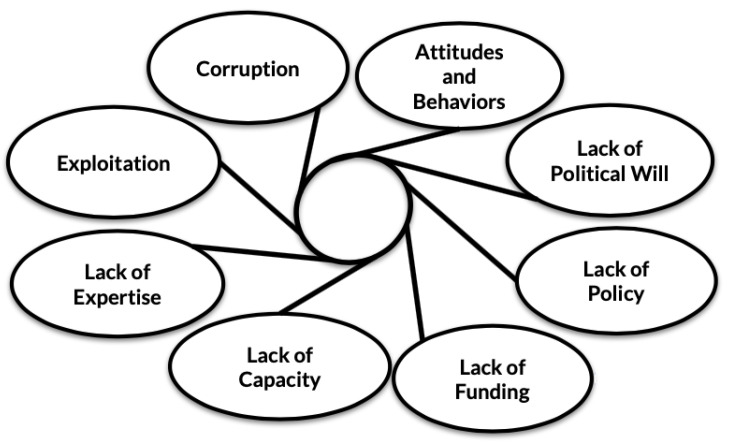
The interrelatedness of identified limitations impeding the proper disposal of confiscated live animals.

The interviews revealed there to be eight categories of barriers to the proper care and disposal of confiscated live animals in Southeast Asia. These are (1) political will, (2) policy, (3) funding, (4) capacity, (5) expertise (6) attitudes and behaviours, (7) exploitation, and (8) corruption. The limitations are related and have the ability to impact each other. For instance, wildlife confiscation authorities are predominantly government institutions, meaning funding priorities are largely dictated by political will (Figure 2).

Government has a key role in limiting the proper disposal and care of confiscated wildlife. A lack of political will is a major barrier preventing adequate funds from being included in disposal budgets. Lack of political will leads to a lack of effective legislation and policy with inadequate protocols and weak enforcement. Without adequate financial resources management is unable to provide sufficient capacity and expertise. These limitations create an avenue for wildlife exploitation in an attempt to make up for gaps in required resources.

The list of identified limitations has consequences for the ‘disposed’ live animals. For example, limited funding results in unsuitable, poorly managed captive facilities that do not meet the welfare needs of wild animals kept there. Exploitation and corruption were recognised as both a limitation to achieving suitable care for confiscated animals and as a consequence of improper management. Concerns have been raised that some captive facilities engage in exploitation and corrupt practices by abusing their legitimate status in order to traffic animals through the use of fraudulent paperwork and/or animal laundering. The lack of accountability allows for engagement in exploitative and corrupt activity by fraudulently using a legitimate business for economic gain. In the absence of adequate funding, even legitimate establishments have turned to morally questionable business models, many involving tourism, to support confiscated wildlife operations—they teeter on what constitutes justifiable subsidies and exploitation. 

Interviews from this study supported CITES data listing zoos as the most common captivity option for confiscated live animals [22]. This opens pathways for exploitation considering zoos are not generally concerned with rehabilitating and releasing animals. As the first disposal choice for many confiscation authorities, zoos are in a position to select only popular species before the animals are taken to a sanctioned rescue centre. The hierarchy of perceived species importance impacts proper disposal management decisions. This research found that the disposal methods chosen for less popular and commonly traded animal species are more frequently animal killings and/or unsanctioned releases, and the disposal method chosen for popular animal species is more frequently captivity, even when that may not be the best disposal option for those animals. 

With insufficient political will to increase funding for legitimate rescue centres, wildlife tourism is relied upon and has been described as a necessary evil. When looking at tourism’s effect on captive wildlife, findings confirm past studies that found an increase in tourists seeking more ethical animal tourism activities [59,70]. In response to the new ‘ethical tourism’ market, rescue centres are emerging across Southeast Asia [71,72], many of which are illegitimate. They put legitimate centres under immense pressure to comply with the demand for animal interactions rather than operating in the animals’ best interest. This trend is magnified in regions where animals have become totemic symbols of countries, for example, elephants and tigers in Thailand and orangutans in Indonesia and Malaysia. Direct interactions with wildlife are sought as part of the exotic cultural experience, but unfortunately, this trend has created a new exploitative market for wildlife. This is not to discount that wildlife tourism experiences have the potential to positively impact public awareness if executed properly [61,73]. Presently, however, this new branch of tourism seems to have more opportunities for inflicting harm and raises the concern that confiscated live animals are being exploited for profit.

The high demand for confiscated animal care has led to governments allowing ad-hoc and illegitimate rescue centres to proliferate. Several participants explained how their country allows private rescue centres to house confiscated animals because the government is not committed to providing adequate resources to manage their own rescue centres. The costs of caring for captive animals are significant, however, one participant explained that faux-rescue centres are able to make a profit. This can be done by charging entry fees, using and/or charging volunteers instead of paying labour, buying substandard food, and not providing adequate veterinary care or environmental enrichment for animals. ‘Back door sales’ were also mentioned alluding to trafficking of animals as an additional revenue source. This unregulated and new form of exploitation takes advantage of naive tourists, and confiscated animals that should be provided with a legitimate, protective shelter. 

The IUCN have reported that releasing confiscated animals back to the wild is not often done [40]. This may be accurate if it refers strictly to properly managed reintroductions, but it has been indicated that releases are happening more frequently than previous research suggests. One possible explanation for this variance between this study’s findings and the CITES survey data is that the majority of Southeast Asian countries did not participate in the CITES survey. Accounts from interview participants indicated that releases done without proper protocols were fairly commonplace. Authorities, therefore, may be unaware of the actual number of releases or they may not want to concede that many releases are unwarranted.

Participants were aware of the extensive protocols involved in releasing rescued animals and speculated that, if funding was in place to support it, attempts at successful releases would be possible. Releasing confiscated animals to their native habitat has the potential to be a powerful conservation tool if implemented correctly [47,74] and could ultimately produce population-level benefits [48]. Pre-release rehabilitation and post-release monitoring are essential components for this method to be successful [61]. At this time, the current system does not always allow for properly planned releases due to a number of limiting factors including ecological risks and significant resource barriers [25,75] which are already limited as this study affirms. If proper release practices could be implemented, they create an opportunity to return a significant number of endangered animals to the wild that may have otherwise been killed, kept in captivity, or lost to the trade. 

### 4.1. Recommendations

#### 4.1.1. Wildlife Seizure Management

This study exposes the troubling reality of a key part of the illegal wildlife trade in Southeast Asia. Many problems stemming from the mismanagement of confiscated live animals were reported, indicating an urgent need for controls and supportive mechanisms. Developing a stronger chain of custody and recordkeeping techniques with advanced data collection methods will be critical in making sure protocols post-seizure are sanctioned and properly delivered. Also, the management of captive facilities need to be held accountable through licensing, permitting, regulations, and regular inspections. NGOs, government agencies, and the media can be supportive by focusing less attention on the seizure itself and by prioritizing awareness on demand reduction and stressing prosecutions from seizures. Seizures should remain a prioritised strategy for disrupting trade. There are only a limited number of authorities that can legitimately confiscate live traded wildlife. By establishing training and support that enables authorities to initiate the seizure in exporting countries, there will be a greater chance for the animals to be released in their native ranges. Current practices of short-term training, capacity building, and knowledge sharing need to be redefined with funding allocated toward transparent programs that involve local communities and promote long-term goals.

#### 4.1.2. Legislative Support

Stronger legislation is fundamental to protecting illegally traded wildlife. Judicial support is important especially concerning live evidence requirements. Live evidence requirements are viewed as detrimental since they often prohibit the release of wild animals when suitable. The adoption of new evidentiary standards that do not involve the detainment of live animals, such as photographic, DNA and/or tissue evidence, should be considered. If live animal evidence remains a requirement, it is suggested that in cases involving a large number of confiscated specimens, a small proportion of the animals should be sufficient to meet this judicial requirement, which would allow remaining animals to be rehabilitated and released more swiftly. Because funding is a central limiting factor preventing well-managed disposal methods, it is recommended that states adopt or strengthen legislation to levy fines on convicted criminals to recoup costs associated with confiscation and disposal of live animals [8,76].

#### 4.1.3. Enhanced Political Will

Enhancing political will to facilitate progressive reforms is key to legislative support and appropriate funding. Decision-makers can consider the substantial value of wildlife considered as natural capital. Many Southeast Asian nations are heavily reliant on tourism, and controversies related to the illegal wildlife trade may affect this important income stream. Furthermore, the United Nations Sustainable Development Goals include respect for life below water and life on land. Decision-makers play a key role in the future of the viability of threatened species. Biodiversity is a fundamental good with potential consequences for the environment and human health. Illegally traded wild animals are sentient beings with intrinsic value, which should be respected by enlightened nations in the twenty first century. All of these considerations suggest that wise decision-makers and governments would legislate and resource appropriately to regulate the illegal wildlife trade, including the proper care and disposal of confiscated live animals.

#### 4.1.4. Demand Reduction

Another essential yet challenging component of the illegal wildlife trade is to reduce demand for live animals. Much of the trade is the result of harmful perceptions and a lack of knowledge surrounding the live animal trade. Consumers, including tourists, have limited understanding of the reality and impacts of the trade on individuals and species. Public awareness campaigns coupled with stronger government regulation can help decrease demand for exotic animals on an individual level. With respect to tourism practices, studies suggest meeting the needs of wildlife is better achieved when programs involve visitors as conservation partners by communicating the reasons behind constraints imposed on interactions with animals [77].

#### 4.1.5. Global Participation

The frequency and intensity of confiscations from the illegal wildlife trade is much higher in some regions than in others; however, cost and responsibility should not be a disparate burden. Southeast Asia, for example, has the highest levels of reported confiscations [9], but significant economic constraints limit the ability of these nations to manage them well. The top need in the region for tackling the illegal wildlife trade is expanded international cooperation and commitments [78]. In this research, one participant made a profound comparison between displaced peoples due to war and conflicts and the displaced animals due to the wildlife trade. When human refugees arrive on the shores of other countries, there is an expectation for the global community to abide by moral responsibility to provide aid. The same should be done for displaced live animals.

#### 4.1.6. Registry of Rescue Centres

There is an urgent need for proper licensing, registration, and oversight of captive facilities. Mandatory basic standards (i.e., housing, nutritional and enrichment requirements, behavioural training when warranted, etc.), inspections, and appropriate training for staff and volunteers, often need to be introduced. It is also important to require an educational component for public facilities to present to visitors. A governing body to oversee efforts would promote transparency and oversight. It is important to acknowledge the differing capabilities across states and provinces and requirements would need to exist on a scale, without compromising principle. This would allow countries to create achievable benchmarks that would not disincentivise confiscating authorities. A critical step in ensuring that confiscated animals do not continue to be exploited will be to also close illegitimate facilities.

#### 4.1.7. Terminology Change

The word ‘disposal’ is a technical term used by CITES and national governments for confiscation of live specimens. In common parlance, the term is defined as ‘the act of getting rid of something, especially by throwing it away’ [79]. The term ‘disposal’ generally refers to inanimate, i.e., non-sentient beings. The use of ‘disposal’ by CITES and the international conservation community, therefore, objectifies and degrades sentient wild animals. Given that language propagates oppressions [80], the term ‘disposal’ is unlikely to facilitate favourable treatment both at individual and species level in the wildlife trade. 

We propose that CITES and the wider conservation community move to a more positive discourse when referring to illegally traded and confiscated live specimens. This could be a constructive step in enhancing perceptions surrounding this topic and encourage authorities to think differently about how they respond to live animal confiscations. ‘Management of’, ‘treatment of’, and ‘custody of’ illegally traded and confiscated live specimens are potential alternatives. 

Similarly, the common misuse of ‘euthanasia’ can legitimise animal killing that does not meet the definition of euthanasia which requires the killing to be both humanely conducted and in the best interests of the animal [45,46]. This may contribute to the overuse of killing as a management tool. Accordingly, animal killing should be accurately described as such, with ‘euthanasia’ reserved for killing that is genuinely in the best interests of the animals concerned.

## 5. Conclusions

This research adds to the limited knowledge regarding the ‘disposal’ of confiscated live animals, including associated limitations and the impacts of improper management on animals. It calls attention to the importance of human attitudes and behaviour, which play an influential role in the relations between barriers to the proper disposal, and their consequences for welfare and conservation. If one limitation is strained, it can cause a ripple effect, making resolutions more challenging. On the other hand, if one or more influences are mitigated, there is potential for improvement across the spectrum of limitations.

This research has raised questions in need of further investigation. The qualitative approach could be applied to systems in other regions and countries to gain more comprehensive insight on the topic. There is a notable lack of literature on the disposal of confiscated live animals, highlighting the need for academic research to provide verifiable detail with regard to exploitation, abuse, and corruption as well as mismanagement, at this stage in the wildlife trade chain. Successful improvement efforts will be largely dependent on societal shifts, political will, and the development of a framework for collaboration between regional and international agencies. It is clear that a ‘one size fits all’ approach to tackling proper management strategies for confiscated live animals is unlikely to be effective, and that individual, case-dependent solutions must be developed for long-term sustainable solutions for rescued animals.

The conversation on disposal of confiscated live animals is growing, but there is still a long way to go. The prohibitive limitations described in this study create severe consequences that threaten global conservation objectives and undermine the ethical treatment of wildlife. Because of its prominence within the illegal wildlife trade, Southeast Asia is a region where progress would produce substantial impacts across the globe. Reshaping mindsets and harmful perceptions are challenging, but these may be the most effective ways to ensure better outcomes for recovered animals. Confiscated live animals will have already beaten the odds by being intercepted by authorities and should not be met with further exploitation and harm. They deserve the best possible chance to survive the trade.

## Figures and Tables

**Table 1:** Interview guide.

Seven Questions Used as a Guide for Interviews
1. What are the limitations impeding proper disposal of live animal confiscations in your region/country?
2. In your opinion, what can be done to improve the handling/disposal of live animal confiscations in your region/country?
3. In a recent CITES survey, more than 30% of participating CITES parties identified their country as having no legislation in place for the disposal of confiscated live animals. In your opinion, what are the barriers preventing countries from including disposal legislation?
4. In which ways are captive facilities impacting confiscated live animals?
5. Describe any cases you are familiar with where confiscated animals were not handled appropriately.
6. Describe how tourism and/or visitors impact captive care facilities in your region/country
7. To the best of your knowledge, what are the captive care facilities in the region/country approved to take confiscated animals and how would you describe their management structure/business model?

**Table 2:** Limitations to the proper ‘disposal’ and care of confiscated live animals categorised based on interviews.

**Limitation**	**Examples of Discourse Used by Participants**
Political Will	lack of inertia, political motivation; concerns related to government follow-through, commitment, inactivity, government perspective; will of enforcement
Policy	concerns related to legislation, procedures, plans of action, protocols, enforcement mechanisms, accreditation, legal requirements, guidelines, governance
Funding	costs related to disposal method, operations, seizures, expenses; budget; any constraint from financial limitations; acknowledgment of regional funding limitations/the need for economic resources
Capacity	inadequate space, insufficient staffing, lack of technical mechanisms, weak data collection, recordkeeping, spatial issues, and equipment; number of animals
Expertise	concerns related to staff education levels, limited knowledge, limited personnel training, improperly trained personnel, educational constraints, lack of learning/training opportunities; acknowledgment of the need for experts, training, educational improvements
Attitudes and Behaviours	detrimental perspectives and viewpoints from community, institutions, and individuals; harmful ego and ego-dominated behaviours; public and institutional perceptions; cultural views negatively affecting management; concerns related to harmful trends
**Limitation and Consequence**	**Examples of discourse used by participants**
Exploitation	the act of making use of and/or benefiting from confiscated animals; harmful for-profit schemes, purchasing animals, acquiring animals; illegitimate establishments
Corruption	dishonest or fraudulent conduct by those impacting the management of confiscated animals; bribery; illegal misconduct; crime

**Table 3:** Summarised limitations to the proper disposal of confiscated live animals based on participant acknowledgment. Participants are labelled C1-C18 to ensure confidentiality and are grouped according to their professional affiliation: non-governmental organization (NGO), NGO run rescue centre (NGO Rescue Centre), government run rescue centre (Govt Rescue Centre), government (Govt), and Academia.

Affiliation	Participant	Funding	Capacity	Expertise	Exploitation	Attitudes	Policy	Political Will	Corruption
NGO	C1	×		×	×			×	
NGO	C2	×	×	×	×	×	×	×	×
NGO	C6	×	×	×	×	×	×	×	×
NGO	C8	×	×	×	×		×		
NGO	C10	×	×	×		×		×	
NGO	C12	×	×		×	×			×
NGO	C13	×	×	×	×	×	×		
NGO	C14	×	×	×	×	×	×		
NGO	C15	×	×	×	×	×	×	×	×
NGO	C18	×	×	×	×	×	×	×	×
NGO Rescue Centre	C16	×	×		×	×	×	×	
NGO Rescue Centre	C17	×	×	×	×		×	×	×
Govt Rescue Centre	C4	×	×	×	×	×	×		×
Govt Rescue Centre	C9	×	×	×	×			×	
Private Rescue Centre	C5	×	×	×	×	×	×	×	×
Govt	C3	×	×	×	×	×	×	×	×
Govt	C7	×	×	×	×	×			×
Academia	C11	×	×	×				×	

## Data Availability

Data may be available on request from the corresponding author. The data are not publicly available due to ethical and privacy restrictions.
